# Pan-cytokeratin immunoexpression in Wilms' tumors: a simple approach for understanding tumor epithelial differentiation

**DOI:** 10.1590/S1516-31802004000400011

**Published:** 2004-07-01

**Authors:** Simone Treiger Sredni, José Ivanildo Neves, Beatriz de Camargo, Otávia Luiza Damas de Caballero, Fernando Augusto Soares

**Keywords:** Wilms' tumor, Immunohistochemistry, Cytokeratin, Kidney, Kidney neoplasms, Tumor de Wilms, Imunohistoquímica, Citoqueratinas, Rim, Neoplasias renais

## Abstract

Wilms' tumor is one of the most common solid tumors in children and is an interesting model for understanding the pathogenesis of embryonal tumors. Cytokeratins are intracellular fibrous proteins present in tissue of epithelial origin. The immunoexpression of the pan-cytokeratin AE1AE3 was studied in paraffin-embedded tissue sections from 24 Wilms' tumors (1 2 with nephrogenic rests) and also tissue samples from 15 corresponding normal kidneys, to evaluate epithelial differentiation in the genesis of Wilms' tumor. We observed that the intensity of the expression of AE1AE3 in the epithelial component of Wilms' tumors directly correlated with the degree of maturity of the epithelial structures correspondent to the collecting ducts.

## RESUMO

Os tumores de Wilms são alguns dos mais freqüentes tumores sólidos da infância e reconhecidamente urn modelo para a compreensão da patogênese dos tumores embrionários. As citoqueratinas são proteínas intracelulares presentes em tecidos de origem epitelial. Estudamos a imunoexpressão da pan-citoqueratina AE1AE3 em 24 tumores de Wilms, dentre ao quais 15 continham também tecidos renais não-neoplásicos e 12 apresentavam restos nefrogênicos em blocos de parafina, para avaliar a diferenciação epitelial no desenvolvimento dos tuniores de Wilms. Observamos aumento na intensidade de expressão de AE1AE3 no componente epitelial dos tuniores de Wilms diretamente relacionado ao grau de maturação das estruturas epiteliais correspondentes aos ductos coletores.

## INTRODUCTION

Because of the resemblance of childhood embryonic tumors to the corresponding fetal tissue, such tumors are good indicators of the relationship between cancer and the fetal developmental process. Wilms’ tumor is an embryonic renal neoplasm and, despite its low frequency, it has proven to be a good model for understanding the pathogenesis of human neoplasia. It is presumed that Wilms’ tumor originates from abnormalities in renal histogenesis, and study of its histology gives evidence that its pathogenesis is closely related to the developmental biology of the kidneys. It is often associated with additional developmental abnormalities in the genitourinary system. The most common of these abnormalities is the persistence of the nephrogenic rests. The generic term “nephrogenic rest” is applied to the persistent embryonic remnants in the kidney that are apparent precursors of Wilms’ tumor. They can occasionally be found in normal neonatal kidneys but usually regress during the first year of life.^1^

It is believed that Wilms’ tumor arises from malignant transformation of renal stem cells. The classic three-phase Wilms’ tumor contains variable quantities of blastemal, epithelial and stromal elements, which are believed to be differentiated products from a common clone of the primitive renal stem cell.^2^

Cytokeratins are members of the intermediate filament protein family. They consist of more than 20 different subclasses grouped according to their molecular weights or pattern of expression through simple or complex epithelium. They can be mapped using a battery of monoclonal antibodies, via immunohisto- chemical methods. For scanning purposes, several of these antibodies can be combined in the form of “cocktails”. AE1AE3 is one of these cocktails that recognize basic and acidic cytokeratins. It is widely used to distinguish epithelial from non-epithelial tumors.

Successful diagnostic use of the expression of certain antigens by embryonic tumor cells depends on the evolution of antigen expression during tumor differentiation. Increasing levels of cytokeratin expression accompany epithelial differentiation in tumors. The diagnostic use of an antibody must be related to the clinical data and morphological aspects of the tumor, because tumor cells may increase the expression during the differentiation process.

## MATERIAIS AND METHODS

24 cases of Wilms’ tumor and 15 tissue samples from the corresponding normal kidneys were retrieved from the archives of the Pathology Department of Hospital do Cancer (Sao Paulo, Brazil). All samples were studied by optical microscopy and immunohistochemistry.

The reactivity to pan-cytokeratin was detected via specific monoclonal antibodies (AE1AE3, Dako) that had previously been optimized at a dilution of 1:200, using standard immunohistochemical methods on paraffin 5-μm formalin-fixed, paraffin-embedded tissue sections. Epitope retrieval was performed by placing slides in a Coplin jar containing 0.01 M citrate buffer (pH 6.0) and microwaving for 15 min on the high setting in a 900-watt microwave oven. The sections were stained for AE1AE3 using an automated stainer (i6000™ Consolidated Staining System, Instrument Model 1.0, with i6000™ Consolidated Staining System software version 1.1, BioGenex Laboratories, Inc). The primary antibody binding to tissue sections was viewed using the avidin-biotin-peroxidase method. Sections were counterstained using hematoxylin. Appropriate positive and negative controls were utilized.

## RESULTS

**Figure 1:**
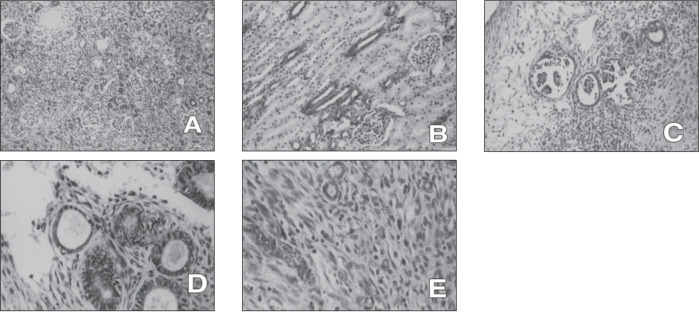
A-Nepbrogenic rests expressed positivity for AΕ1ΑΕ3 within the tubular structures but not within glomeruli (200x);B-In the normal kidneys, the collecting ducts were positive and glomeruli were negative for AE1AE3 (400x);C, D and E- The mature duct structures showed regular and complete immunoreactivity for AΕ1ΑΕ3-The more immature the epithelial structure was, the more irregular and incomplete the staining pattem became (C,D and E all 400x).

Histological examination of the slides showed a range of morphological features. Among the 24 Wilms’ tumor specimens, there were 18 three-phase tumors with blastemal, stromal and epithelial components and 6 tumors without epithelial differentiation, consisting of various pioportions of blastemal and stromal components. Among the cases with epithelial differentiation, 17/18 showed immature structures like abortive tubules, rosettes and microcysts, 16/18 showed more mature structures like tubules and glomeruli and 15/18 had both immature and mature structures. Nephrogenic rests were seen in 12 cases. All the nephrogenic rests expressed a mature pattern of AE1AE3 immunostaining in the tubular structures but not within glomeruloid structures (Figure 1A). The same features observed within the nephrogenic rests were observed within the 15 corresponding normal kidneys analyzed: tubules were positive and glomeruli were negative for AElAE3 (Figure 1B).

The epithelial differentiation in the tumors was accompanied by distribution of the immunoreactivity to the individual elements. All of the mature duct structures (16/16) showed immunoreactivity for AE1AE3 involving the epithelial cells lining these structures. The more immature the epithelial structure was, the more irregular the staining pattern became (Figures 1C, 1D and 1E).

Only three out of the 18 tumors that had epithelial differentiation did not express cyto-keratins in the epithelial component. All of these had immature epithelial structures alone.

## DISCUSSION

During normal nephrogenesis, some cells from the metanephric mesenchyme begin to show epithelial characteristics and organize to form the epithelial renal vesicle. The vesicle elongates and twists to form the functioning nephron. The mature kidney consists of epithelial nephrons and collecting ducts.^3,4^

Looi and Cheah (1993)^5^ demonstrated, in nine Wilms’ tumors, that the epithelial cells of maturing tubular structures showed epithelial membrane antigen positivity; immature tubular structures were epithelial membrane antigennegative; and primitive glomeruloid structures were epithelial membrane antigen-negative and vimentin-positive. It is usually recognized that epithelial membrane antigen and cytokeratins 8 and 18 detected by CAM 5.2 show positive staining of tubular structures.^5^

According to Gilbert,^6^ the kidney is thought to originate through a complex set of interactions between epithelial and mesenchymal components of the intermediate mesoderm. The two intermediate mesodermal tissues (the ureteric bud and the metanephrogenic mesenchyme) interact and reciprocally induce each other to form the kidney. The ureteric buds, which are epithelial branches, induce the metanephrogenic mesenchyme to aggregate and cavitate to form the renal tubules and glomeruli, while the ureteric buds become the collecting ducts.

In the present study, the immature epithelial structures like rosettes, microcysts and abortive tubules showed an irregular immunostaining pattern. The more developed the structure was, the more intense and well defined the AE1AE3 immunostaining pattern became. The glomeruloid bodies were always negative.

Among the nephrogenic rests, it was observed that the tubules were positive and the glomeruli were negative for this antibody, as was found among the normal corresponding kidneys analyzed.

Epithelial differentiation towards collecting ducts within the tumors was accompanied by increasing expression of cytokeratin AE1AE3. The negativity for cytokeratin AE1AE3 within the glomeruli seems to be due to its different embryonic origin, since they are derived from the metanephrogenic mesenchyme.

We believe that the next step in this study will be to test the different cytokeratins separately, so as to have better correlation between morphology, kidney structure development and Wilms’ tumor oncogenesis.

## CONCLUSION

We found increased immunoexpression of AE1AE3 in the duct structures of the epithelial component of Wilms’ tumors that was directly correlated with their morphological maturity and embryonic origin. This experiment seems to be a simple approach towards studying abnormal nephrogenesis and a key for further understanding of the genesis of Wilms’ tumor.

## References

[1] Beckwith JB, Kiviat NB, Bonadio JF (1990). Nephrogenic rests, nephroblastomatosis, and the pathogenesis of Wilms’ tumor. Pediatr Pathol.

[2] Zhuang Z, Merino MJ, Vortmeyer AO (1997). Identical geneticchanges in different histologic components of Wilms’ tumors. J Natl Cancer Inst.

[3] Ekblom P (1981). Determination and differentiation of the nephron. Med Biol.

[4] McCrory WW (1983). Normal organogenesis of the human kidney. Prog Clin Biol Res.

[5] Looi LM, Cheah PL (1993). An immunohistochemical study comparingclear cell sarcoma of the kidney and Wilms’ tumor. Pathology.

[6] Gilbert SF, Gilbert SF (2003). Developmental Biology.

